# Electrospun Poly-ε-Caprolactone Nanofibers Incorporating Keratin Hydrolysates as Innovative Antioxidant Scaffolds

**DOI:** 10.3390/ph17081016

**Published:** 2024-08-01

**Authors:** Naiara Jacinta Clerici, Aline Aniele Vencato, Rafael Helm Júnior, Daniel Joner Daroit, Adriano Brandelli

**Affiliations:** 1 Laboratory of Nanobiotechnology and Applied Microbiology, Institute of Food Science and Technology, Federal University of Rio Grande do Sul, Porto Alegre 90000-000, Brazil; naiaraj.clerici@gmail.com (N.J.C.); aline_vencato@hotmail.com (A.A.V.); 2 Postgraduate Program in Environment and Sustainable Technologies, Campus Cerro Largo, Federal University of Fronteira Sul, Cerro Largo 97900-000, Brazil; daniel.daroit@uffs.edu.br

**Keywords:** antioxidant activity, biocompatible materials, keratin, polymeric nanofibers

## Abstract

This manuscript describes the development and characterization of electrospun nanofibers incorporating bioactive hydrolysates obtained from the microbial bioconversion of feathers, a highly available agro-industrial byproduct. The electrospun nanofibers were characterized using different instrumental methods, and their antioxidant properties and toxicological potential were evaluated. Keratin hydrolysates (KHs) produced by *Bacillus velezensis* P45 were incorporated at 1, 2.5, and 5% (*w*/*w*) into poly-ε-caprolactone (PCL; 10 and 15%, *w*/*v* solutions) before electrospinning. The obtained nanofibers were between 296 and 363 nm in diameter, showing a string-like morphology and adequate structural continuity. Thermogravimetric analysis showed three weight loss events, with 5% of the mass lost up to 330 °C and 90% from 350 to 450 °C. Infrared spectroscopy showed typical peaks of PCL and amide bands corresponding to keratin peptides. The biological activity was preserved after electrospinning and the hemolytic activity was below 1% as expected for biocompatible materials. In addition, the antioxidant capacity released from the nanofibers was confirmed by DPPH and ABTS radical scavenging activities. The DPPH scavenging activity observed for the nanofibers was greater than 30% after 24 h of incubation, ranging from 845 to 1080 µM TEAC (Trolox equivalent antioxidant capacity). The antioxidant activity for the ABTS radical assay was 44.19, 49.61, and 56.21% (corresponding to 972.0, 1153.3, and 1228.7 µM TEAC) for nanofibers made using 15% PCL with 1, 2.5, and 5% KH, respectively. These nanostructures may represent interesting antioxidant biocompatible materials for various pharmaceutical applications, including wound dressings, topical drug delivery, cosmetics, and packaging.

## 1. Introduction

Keratins are fibrous proteins present in animal tissues and epidermal appendages. Large amounts of these recalcitrant proteins are found in hair, wool, feathers, horns, hooves, and nails [1]. Feathers, composed of about 90% (*w*/*w*) keratin, are abundant by-products generated by the poultry industry. As a hard-to-degrade solid by-product, feathers represent a challenge for proper management, and environmental risks are associated with their incorrect disposal. Eco-friendly solutions postulate feathers as raw materials with great biotechnological potential [2,3].

Microbial processing, for instance, offers a great possibility for feather valorization. As a protein-rich material, feathers are investigated as a substrate for microbial growth to obtain proteolytic enzymes and bioactive protein hydrolysates [3,4]. *Bacillus velezensis* P45, for instance, is an efficient feather-degrading bacterial strain, and keratin hydrolysates produced by this bacterium display interesting antioxidant properties [5]. Physical and chemical processes have been proposed to extract keratins from by-products, aiming to formulate nanostructured materials. However, few studies apply biological methods for keratin extraction, or the use of microbial keratin hydrolysis for such a purpose [4,6]. This highlights the opportunities of combining the microbial ability for feather degradation and nanotechnology to develop new biomaterials that might be of importance for various industrial sectors. However, the addition of keratin hydrolysates obtained through the microbial bioconversion of feathers into nanofiber scaffolds has not been investigated. Some studies describe the manufacture of electrospun fibers containing keratin, but essentially obtained using chemical extraction methods, such as the reductive chemistry of bovine hooves [7], the sulfitolysis of human hair [8], and wool deoxidation [9], which are very different from the method reported here.

The most common method applied to create nanofibrous structures is electrospinning. In this technique, a natural or synthetic polymer is initially dissolved in a highly volatile (therefore rapidly evaporating) solvent. The obtained solution is then exposed to a high voltage, yielding the dry polymeric fibers [10]. The electrospun nanofiber membranes are highly porous, presenting a high specific surface area and favorable mechanical features. Structurally, nanofibrous membranes prepared by electrospinning natural proteins are comparable to the natural extracellular matrix, suggesting applications in the biomedical field [11].

However, the electrospinning processing of keratins is quite difficult. In fact, due to their low molecular mass (about 15 kDa and 25–60 kDa for feather and mammalian keratins, respectively), keratin solutions usually display low viscosities, which might result in electrospraying failures, producing droplets or crimped fibers. Such problems might be circumvented by mixing keratins with another polymer and/or through reinforcement with suitable fillers [12,13].

In this sense, diverse synthetic polymers have been used to improve the electrospinning properties of keratins [14]. Particularly, poly-ε-caprolactone (PCL) is a non-toxic, biodegradable, and biocompatible hydrophobic polymer with convenient mechanical properties. Furthermore, the production of nanofiber membranes with PCL through electrospinning is facilitated due to its high molecular weight and high viscosity [15]. PCL nanofibers are materials with great nanotechnological potential due to the high ratio between their surface and volume, allowing high loading capacities that might be used to deliver bioactive substances [16,17] and food packaging applications [18,19]. Moreover, PCL nanofibers are described as potential wound dressing materials [17,20], as a nanoplatform for the controlled administration of long-acting drugs for cancer treatment [21,22], and for dermatological skincare applications [23]. Despite the fact that this polymer has been largely used for the encapsulation of conventional drugs, antimicrobials, and bioactive plant extracts, there is a lack of studies on PCL nanofibers as a carrier for bioactive protein hydrolysates or peptides. Thus, the development of PCL nanofibers incorporating bioactive keratin hydrolysates addresses a current demand in the pharmaceutical area for the development of innovative bionanomaterials [24,25].

Antioxidant scaffolds may have varied pharmaceutical uses, including drug delivery platforms, chronic wound healing, soft tissue regeneration, and cosmetical applications. The incorporation of antioxidant biomaterials into widely used polymers can also be useful for stabilization and functionalization purposes [26]. Antioxidant scaffolds prepared with herbal extracts of *Dracaena cinnabari* and *Melilotus officinalis*, were proposed as curatives for skin lesions and wound dressing [20,27], since reducing the oxidative stress may be beneficial to ameliorate healing processes. Moreover, antioxidant nanofibrous scaffolds can be useful materials for skin tissue engineering, transdermal patch, and oral mucoadhesive supplies [28].

In this context, feather keratin hydrolysates were produced through bioprocessing with the keratinolytic strain *B. velezensis* P45 [29]. Then, the electrospinning process was applied to manufacture polymeric PCL nanofibers incorporating bioactive keratin hydrolysates (KHs) as a strategy to functionalize nanofibers for possible biological applications.

## 2. Results and Discussion

This study envisaged the incorporation of an antioxidant keratin-based hydrolysate into polymeric nanofibers. PCL nanofibers incorporating KH were successfully produced through electrospinning. The obtained nanofibers were initially characterized in terms of their morphological, thermal, and mechanical properties, and then their antioxidant and hemolytic activities were assessed.

### 2.1. Thermogravimetric Analysis

**Figure 1:**
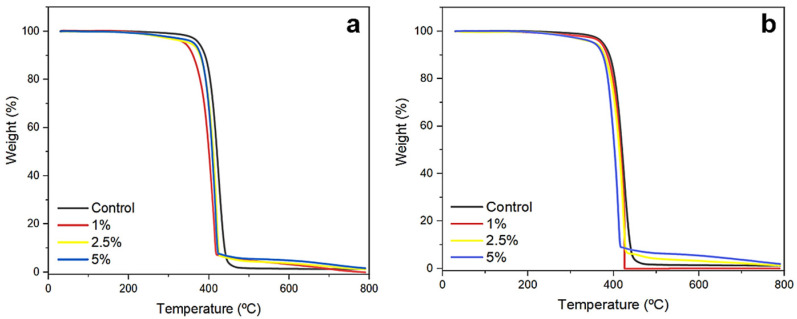
Thermogravimetric analysis of nanofibers produced with PCL and KH. Samples of 10% PCL nanofibers (**a**), and 15% PCL nanofibers (**b**) functionalized with 1%, 2.5%, and 5% KH were subjected to these analyses.

TGA was performed with fabricated nanofibrous materials to determine its thermal stability. The TGA spectrum provides information about weight loss (%) as a function of temperature (°C). The weight loss was minimal in the range 30–100 °C, where water evaporation would theoretically occur [13]. Good thermal stability was observed at 225 °C, with a weight loss of about 5% for all nanostructures (Figure 1). Similar thermograms to those acquired in this work were presented in other studies with PCL-based materials, where it was observed that thermal degradation initiated at around 330–360 °C and proceeded as a single step [30,31]. A similar behavior was reported for PCL/ethylcellulose nanofibers containing natamycin and transcinnamic acid, where weight losses mainly occurred from 350 to 450 °C [32]. Furthermore, those authors had *T*_max_ values close to 400 °C, which corroborate the data observed in this study (Figure 1). A *T*_max_ of 404 °C was also observed for PCL-gelatin nanofiber membranes [33].

Data from the literature reinforce the thermal stability of PCL up to 347 °C, the onset of degradation at 347.19 °C, and the almost complete thermal degradation recorded at 408 °C [34]. In another case, the pure PCL scaffold completely degraded at 417 °C [35], while in this study total mass loss occurred at almost 700 °C. Nanofibers of PCL (12% *w*/*v*) diluted in acetic acid (90%), presented *T*_onset_ at 326.08 °C and *T*_max_ at 399.23 °C, with 1.89% remaining weight at 590 °C [36]. The mass of the nanofibers was virtually extinguished above 700 °C (Figure 1), unlike that observed for PCL fibers formulated with polyacrylic acid and graphene oxide, where 10% of the initial weight remained at 700 °C [37].

About 70% of PCL nanofibers degraded at a temperature of 450 °C, and its thermal decomposition occurred in a single step between 327 and 450 °C, where random polymer chain rupture and the generation of ɛ-caprolactone through depolymerization (unzipping) occurred [38]. Reinforcing the thermograms presented here, PCL exhibited a single-stage thermal degradation, initiating at 346 °C and ending at 430 °C, with a maximum temperature of 390 °C, with 0.5% residue at 650 °C, owing to its synthetic character [39].

### 2.2. Differential Scanning Calorimetry

**Figure 2:**
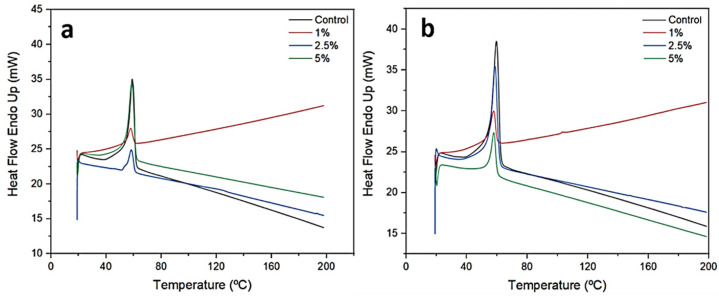
Differential scanning calorimetry analysis of nanofibers produced with PCL and KH. Samples of 10% PCL nanofibers (**a**), and 15% PCL nanofibers (**b**) functionalized with 1%, 2.5%, and 5% KH were subjected to these analyses.

DSC might be applied to investigate the properties of polymer composites, including molecular interactions, miscibility, and the degree of crystallization. In addition, information on the exothermic and endothermic shifts of polymer nanocomposites can be gathered from DSC graphs of temperature (°C) versus heat flow (mW/mg). All of the nanofiber films investigated here had a single transition peak (Figure 2), denoting that the nanofiber components were effectively miscible. Results from the literature also demonstrate DSC curves for PCL nanofibers, where an endothermic peak is noted, indicating the beginning of the fusion process [34,36,40]. From the DSC thermograms, the endothermic peaks can be seen at approximately 60 °C (Figure 2), corresponding to the melting point already described for PCL nanofibers [22].

The *T*m obtained for control nanofibers produced with 10% PCL and 15% PCL (Table 1), was similar to that previously reported for PCL (59.3 °C) and PCL/chitosan fibers (58.19 °C) [36]. Some values found in the literature for PCL (12%, *w*/*v*) were *T*m at 58.73 °C and melting enthalpy (Δ*H*m) at 91.41 J/g [36]. In another study, an electrospun PCL membrane (10%, *w*/*v*) exhibited a *T*m of 63.1 °C, showing a decreased degree of crystallinity and Δ*H*m when nanostarch was loaded into the polymer matrix [41]. Similar phenomena were observed for PCL-KH nanofibers in this study (Table 1). The addition of KH did not greatly impact the melting temperature, and the same was true of the addition of chitosan and capsaicin-loaded alginate to PCL nanofibers (*T*m ranging from 57.3 °C to 59.5 °C), suggesting good miscibility between the materials [22].

The variation in the Δ*H*m of bare polymeric nanofibers as compared with those containing other compounds in the formulation has been described. For example, a decrease from 91.41 to 51.15 J/g was recorded when chlorogenic acid-loaded halloysite nanotubes were included in PCL nanofibers [36]. In this study, the presence of the protein keratin might interfere in the molecular interactions of polymer chains, thereby inducing modifications in the thermal properties of the material [42], such as the melting enthalpy.

### 2.3. Fourier-Transform Infrared Spectroscopy

**Figure 3:**
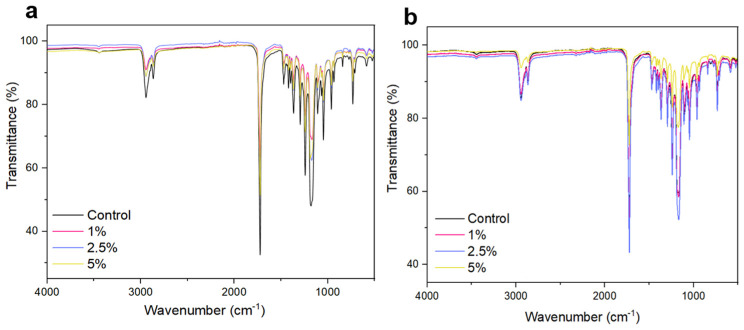
FTIR spectra of nanofibers composed of 10% PCL (**a**) and 15% PCL (**b**). Control (black); 1% KH (pink); 2.5% KH (blue); and 5% KH (yellow).

The FTIR spectra of nanofibers produced with 10% PCL and 15% PCL are presented in Figure 3a and Figure 3b, respectively. The characteristic peaks of PCL were observed at 2943 cm^−1^ referring to the C-H group, and the C=O group at 1740 cm^−1^ which is a typical carbonyl elongation of PCL [34,43,44,45]. PCL shows peaks at 2949 cm^−1^ and 2865 cm^−1^, related to asymmetric and symmetric CH_2_ stretchings, respectively, and the carbonyl band (C=O carbonyl ester groups) is detected at 1727 cm^−1^ [31]. Peaks at 1170 cm^−1^ and 1240 cm^−1^ correspond to symmetrical and asymmetrical stretchings of C-O-C, and the peak at 1293 cm^−1^ indicates the stretching of C-O and C-C [46,47]. Furthermore, the sharp peak at 1242 cm^−1^ is typical of the amide III band (1220–1300 cm^−1^) [43]. The peak at 1042 cm^−1^ was also verified (Figure 3), which is representative of the C-OH stretching [48]. Bands in the range 1480–1580 cm^−1^ can be associated with amide II bands of keratin peptides [49].

The bands in the 1500–900 cm^−1^ range were assigned to the main structure of the PCL polymer, for instance, the bending and stretching vibrations of methylene groups and the gauche and trans isomerization of ester groups, and might indicate shifts in PCL crystallization [50]. The peak at 1025 cm^−1^ was representative of the S-O stretching. The amide I and amide II bands were detected in the PCL-KH fibers, thus indicating the presence of keratin/keratin peptides. Some moderate displacements can be noted in amide II, which can be explained by local interaction between PCL and keratin molecules, through weak hydrogen bonding between carbonyl groups and amides [12,13,51].

The amide I band (1700–1600 cm^−1^) is especially sensitive and largely used to predict protein secondary structures [52]. Thus, such band indicates the existence of proteinaceous components of KH within the nanofibers. The absorption peak at approximately 1700 cm^−1^ and the bands at 1610–1633 cm^−1^ are typical of α-helical and β-sheet structures in keratins [2].

### 2.4. Mechanical Properties

The effects of KH addition on the mechanical properties of the PCL electrospun nanofibers were investigated through the determination of Young’s modulus (*E*), tensile strength (σ), and elongation at break (%). Using 10% PCL composition, Young’s modulus decreased from 42.64 MPa in the nanofibers without KH to 25.93–25.85 MPa in nanofibers with 2.5 or 5% KH, indicating a decrease in the stiffness of the nanofiber mats (Table 2).

A study recently found that fiber diameter interferes with the mechanical properties of PCL nanofibers, and that thinner fibers have much greater stiffness than thicker ones [53]. Pure PCL nanofibers have tensile strength equivalent to 2.586 ± 0.13 MPa [54] and 2.077 MPa [55], similar to the result of the mechanical test presented here (Table 2). It is known that materials with higher elongation at break present improved elastic properties, and a low elastic modulus conveys to materials lower resistance to deformation and higher flexibility [56]. Results with similar Young’s modulus were shown for the fabrication of PCL nanofibers in addition of chitosan methacrylamide (27.6 ± 0.1 MPa) [57].

Much higher values are also reported for chitosan/poly(ε-caprolactone) hybrid nanofibrous mats, corresponding to 268.0 ± 31.82 MPa [58]. The values presented here for the percentage of elongation (199%-2.5% KH/PCL) resemble the mechanical properties of skin tissue, that is, a tensile strength of 1–32 MPa, a Young’s modulus of 2.9–150 MPa, and an elongation at break of 17–207% [59]. Other studies also reported a high elongation at break, in the range of 189.24 ± 6.14 [46].

### 2.5. Scanning Electron Microscopy

**Figure 4:**
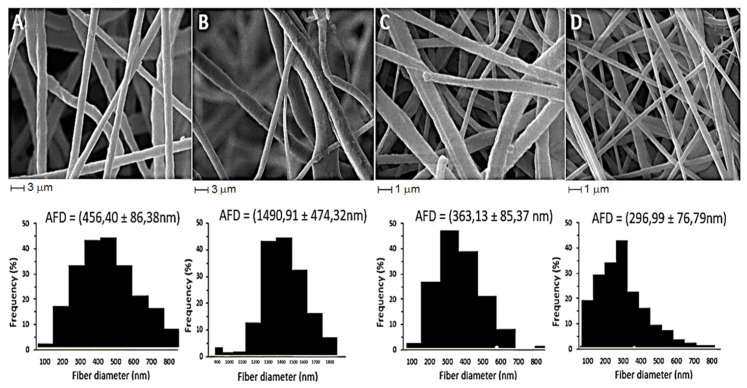
Scanning electron microscopy of 15% PCL nanofibers loaded with 5% bioactive keratin hydrolysate (KH) (**A**) compared with the respective control prepared with only the polymer in the same concentration (**B**). SEM images of 10% PCL nanofibers loaded with 5% KH (**C**) and nanofiber control 10% PCL (**D**). The histograms of the diameter distribution of nanofibers are presented below each SEM image.

SEM analyses revealed that all nanofibers display a morphological appearance free of spheres (polymer clusters) with adequate structural continuity (Figure 4). The nanofibers produced with 10% PCL plus KH demonstrated an increased average diameter (363 nm) as compared to control nanofibers (296 nm). The opposite was noticed for the fibers prepared with 15% PCL, as the addition of KH reduced the mean diameter in comparison with control nanofibers (Figure 4). These values are in agreement with some previous reports of nanofibers produced with 12% PCL showing 1672 nm average diameter [36], whereas 15% PCL fibers showed an average diameter of 1672 nm [60].

Fiber morphology and diameter are strongly dependent on formulation, thus influencing the conductivity and viscosity of the electrospinning solutions, which may explain the different average sizes found in the literature [61,62]. Some values found for PCL nanofiber formulations are 288 nm for 20% PCL [63], 644 nm for 20% PCL with the addition of 20% zein and 6% gum Arabica [64], and 385 nm for 7% PCL [65]. Moreover, depending on the solvent used, the average diameter of PCL fibers might change, for example, from 2.86 μm using chloroform/DMF to 1.36 μm using acetic acid [66].

PCL nanofibers incorporating keratin extracted by chemical reduction (12.5:5 *w*/*w* PCL/keratin) showed an average size of 532 ± 80 nm, while the control PCL nanofibers had a mean diameter of 372 ± 74 nm [67]. Those authors also observed an increase in size when incorporating keratin into nanofibers, in agreement with the results of this study for 10% PCL nanofibers. Variations in the size of nanofibers could be associated with the increased viscosity of the spinning solution owing to the inclusion of keratin (Table 3).

Viscosity values are important since the electrospinning solution needs to form a continuous jet flow towards the collector plate. A very low viscosity is often incompatible with the homogeneous formation of nanofibers, as the degree of entanglement of the polymer chains is small and often causes instability in the jet [68]. Within an appropriate range, an increase in the viscosity of the solution allows obtaining a cone-jet close to the theoretical Taylor cone, reducing the mean diameter of the fibers [15], although very high viscosity interferes with the evaporation of the solvent, resulting in fibers with larger diameters [42]. Moreover, the size and morphology of nanofibers can be influenced by the presence of additives, the applied voltage, and the surface tension of the solution [68]. Thus, the increase in solution viscosity through the addition of KH might explain the approximately five-fold decrease in the mean diameter of 15% PCL nanofibers.

### 2.6. Antioxidant Activities

**Figure 5:**
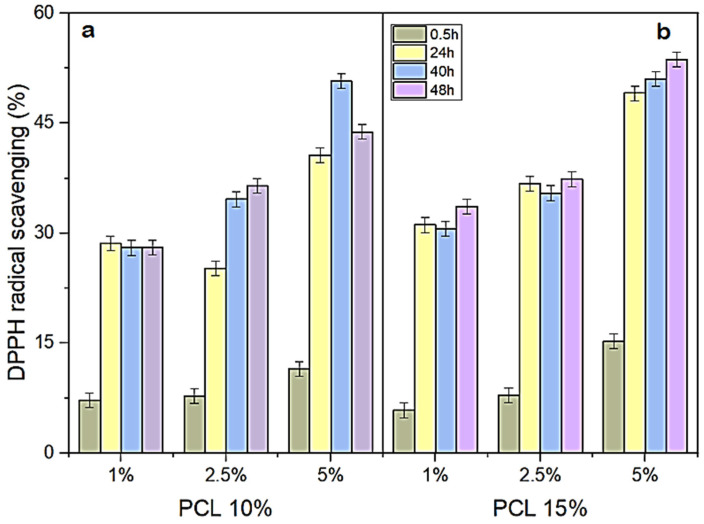
Scavenging of the DPPH radical from nanofibers composed of 10% PCL (**a**) and 15% PCL (**b**). All nanofibers tested were functionalized with different concentrations of bioactive keratin hydrolysate (1, 2.5, and 5%). Data represent the mean ± standard deviation of three independent experiments.

The antioxidant potential of the nanofibers was initially assessed through the scavenging of the DPPH radical, and the results are summarized in Figure 4. The addition of nanofibers directly to the DPPH solution resulted in higher antioxidant activity for the samples incubated for longer periods (24, 40, and 48 h) as compared to that measured at 30 min. Moreover, nanofibers containing 5% KH showed higher DPPH scavenging capability, irrespective of the PCL concentration. The maximum antioxidant activity for 10% PCL + 5% KH formulation was recorded at 40 h (50.7% DPPH scavenging, corresponding to 995.2 µM Trolox equivalents (TEAC)). For 15% PCL + 5% KH nanofibers, a maximum value of 53.7% was observed at 48 h, corresponding to 1035.2 µM TEAC. These values were higher as compared to 30 min (Figure 5). Considering the lack of studies reporting nanofibers carrying antioxidant peptides, this research presents a novelty in the area.

The incorporation of natural biopolymers like gelatin and keratin to PCL fibers provides a complex of good hydrophilicity and cellular affinity. The sustained release of peptide molecules from the nanofibers and their biocompatible characteristics suggest potential for would healing, the delivery of bioactive substances, and tissue regeneration [69,70].

Clearly, the antioxidant activity was greater when 5% KH was incorporated into the nanofibers, and the differences between the formulations containing 1% and 2.5% KH were not remarkable when compared with each other (Figure 5). This suggests a concentration-dependent release of the antioxidant molecules and that at least 5% KH should be added to the formulation to maximize the biological activity. Even for 30 min, which was the shortest incubation time for this experiment, the values were around 15% scavenging for 5% KH, and about 6% for either 1% or 2.5% KH. Supporting the data presented here, a greater inhibition of free radicals was observed for PCL and lignin nanofibers, as a function of the incubation time or lignin concentration [71]. The antioxidant potential of PCL–lignin increased at higher proportions of lignin in the formulation, likely because of the presence of antioxidant phenolic groups. Among all nanofibers, those containing 20% lignin showed the highest DPPH scavenging activity, reaching 97.5% after 48 h. The nanofibers had stable antioxidant activity over a long period, similarly to that observed in this study.

The low antioxidant activity of PCL nanofibers at short incubation times may be related to the polymer nature and degradation profile, resulting in very low release rates. PCL is quite stable, since it has few ester bonds per monomer, which leads to longer degradation and slower release rates [72,73]. In this regard, the nanofibers were evaluated after they underwent an extraction process [74]. The results achieved from the nanofiber extracts showed that all formulations containing KH presented antioxidant activity, highlighting that similar values were observed for nanofiber extracts incubated for 60 min (Table 4) and nanofibers directly incubated for 48 h in the DPPH solution (Figure 5). Thus, it can be concluded that the extraction step served to improve the release of the bioactive molecules contained in the KH.

The second antioxidant test carried out in vitro was the test using ABTS radical. We obtained an increase in activity with the increase in KH from 44.19 to 56.21% (969.78–1240.89 μM TEAC), results using 1% and 5% of KH, as shown in Table 4. The high ABTS radical capture capacity was indicated for active packaging such as chitosan film/esterified chitin nanofibers incorporating anthocyanins extracted from eggplant peel to indicate pork freshness. The use of eggplant peel at 4% caused a 90% elimination of the radical; with 2% of the material, the activity was a little less than 80%, and the activity with chitin alone was 30% [75]. Nanofibers containing phycocyanin showed a 24.68% elimination of the ABTS radical [76], while another study achieved a result of 29.7% for the same test containing phycocyanin derived from the microalgae *Spirulina platensis* [77]. Other bioactive nanofibrous materials achieved a radical scavenging of 29.6% ± 2.6 (12% PCL + 3% curcumin), and the authors suggested as a possible application that biomaterials with antioxidant activity help control oxidative stress in damaged tissues [78].

Nanofibers containing bioactive substances have been proposed as interesting materials for wound dressings, since their antioxidant property would eliminate free radicals and thus decrease inflammatory response [79]. Wound healing is a complex process. In the inflammatory stage, which begins with hemostasis, diverse pro-inflammatory cytokines, proteases, and reactive oxygen species are released. Excessive amounts of the latter can impair wound healing due to, for instance, detrimental effects on fibroblasts, the main cells responsible for collagen synthesis during wound repair [59,69]. Therefore, there is an effort to create dressings that are not only antimicrobial, but also antioxidant materials, which can release bioactives in a controlled and sustained manner. It was found that PCL nanofibers in addition to gelatin eliminated 8.69% of DPPH in 30 min, which may be ascribed to the radical scavenging activity of a gelatin-derived peptide (His-Gly-Pro-Leu-Gly-Pro-Leu) [80]. Here, in 30 min, we obtained 11.48% activity using 5% KH, and increased activity was observed for longer times, suggesting a sustained release of antioxidant activity.

### 2.7. Hemolytic Activity

**Figure 6:**
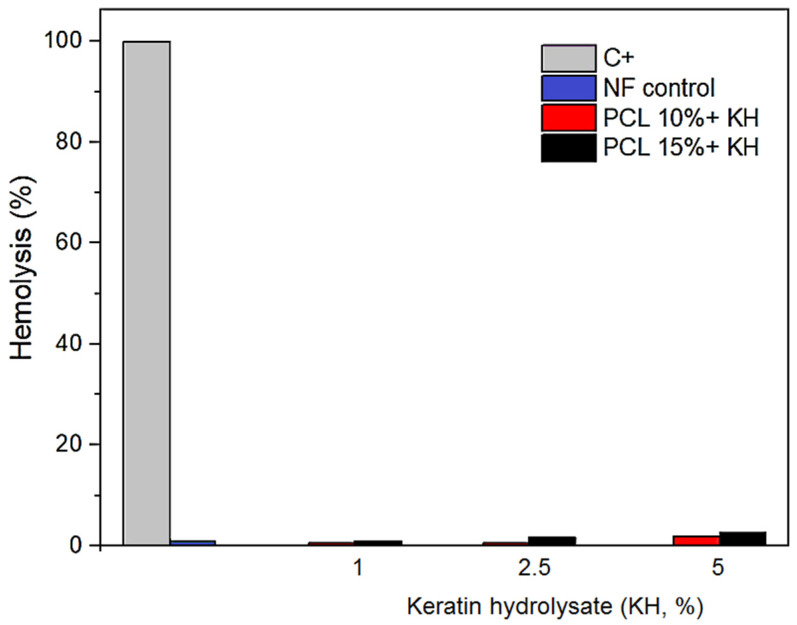
Hemolytic activity evaluation of PCL nanofibers functionalized with bioactive keratin hydrolysate (KH) at different concentrations (1, 2.5, and 5% KH) compared with PCL nanofibers without KH (PCL control). Triton X-100 was used as positive control (C+) and was considered as 100% hemolysis. Results are the means ± standard deviations of three independent experiments.

The blood compatibility of diverse materials is usually assessed through hemoglobin release from erythrocytes, which indicates cell damage. Hemocompatibility is among the crucial criteria for the evaluation of biomaterials, as it can cause detrimental side effects, thus limiting its applicability. The permissible range of hemolysis activity for biomedical materials is 5% [55,81]. The developed nanostructures exhibited a low hemolysis rate (Figure 6). It was verified that hemolysis increased as the content of FH increased; however, the highest hemolytic activity was 2.57% for nanofibers composed of 15% PCL plus 5% KH.

The hemocompatibility of PCL nanofiber mats containing chitosan/CeO_2_ nanoparticles has been previously confirmed, since the hemolytic activity was 0.78%, supporting their use in the healing of wounds in contact with blood cells [55]. Other nanofibrous materials composed of PCL also showed low hemolytic rates, which reinforce its low toxicity [34,35].

The lack of hemolytic activity and antioxidant properties of PCL-KH nanofibers suggest their safe application as drug delivery or wound dressing platforms. The mitigation of oxidative stress is critical to help the process of tissue repair and regeneration. The scavenging of deleterious and over-accumulated reactive oxygen species can be achieved by using antioxidant scaffolds, accelerating healing in tissue engineering applications [26,28,69]. Thus, the nanofibers developed in this study appear to be biocompatible materials that can be beneficial platforms for the delivery of bioactive agents in pharmaceutical applications.

## 3. Materials and Methods

### 3.1. Chemicals

Poly-ε-caprolactone (PCL; average MW 80,000 Da), the 2,2-diphenyl-1-picrylhydrazyl (DPPH) and 2,2′-azino-bis-(3-ethylbenzothiazoline)-6-sulfonic acid (ABTS) radicals, and 6-hydroxy-2,5,7,8-tetramethylchroman-2-carboxylic acid (Trolox) were purchased from Sigma Aldrich (St. Louis, MO, USA). Tetrahydrofuran (THF) and N-dimethylformamide (DMF) were from Merck (Darmstadt, Germany). Sodium chloride (NaCl), monopotassium phosphate (KH_2_PO_4_), and calcium chloride (CaCl_2_) were acquired from Labsynth (São Paulo, Brazil). The chicken feathers were obtained from a private rural property (Guarani das Missões, RS, Brazil). All other reagents were of analytical grade and distilled water was used to prepare all solutions.

### 3.2. Microorganism

The previously isolated keratinolytic bacterium *Bacillus velezensis* P45 was used to produce feather hydrolysates [29]. The strain was obtained from the culture collection of the Laboratory of Biochemistry and Applied Microbiology (ICTA, UFRGS, Brazil). Brain–heart infusion (BHI) agar plates were inoculated and incubations occurred at 30 °C for 24 h. The isolate was kept on BHI agar plates under refrigeration (7 °C) and subcultured periodically during the experiments.

### 3.3. Production of Keratin Hydrolysate (KH)

Feathers were carefully cleaned and soaked in chloroform–methanol (1:1 ratio, *v*/*v*) to remove stains or any grease residue. Clean feathers were cut and added (10 g/L) to a mineral medium (0.3 g/L K_2_HPO_4_, 0.4 g/L KH_2_PO_4_, 0.5 g/L NaCl). After the initial pH was adjusted to 7.0, the medium was sterilized by autoclaving at 121 °C, 105 kPa, for 15 min. Cultivation was conducted in 250 mL flasks containing 50 mL of medium inoculated with 1% (*v*/*v*) of a bacterial suspension of *B. velezensis* P45. Submerged cultivations were carried out at 30 °C, 125 rpm, for 72 h. Subsequently, cultures were centrifuged (10 min, 10,000× *g*, 4 °C), and the supernatants were frozen, lyophilized [82], and then used for nanofiber production.

### 3.4. Nanofiber Manufacturing

PCL was dissolved using a 1:1 (*v*/*v*) ratio of THF:DMF at concentrations of 10% (0.1 g/mL) and 15% (0.15 g/mL). KH was mixed with PCL solutions at three concentrations (1, 2.5, and 5%, based on polymer weight) to generate different polymeric solutions (PS). For electrospinning, a syringe with 3 mL of PS was coupled to an electrospinner (BR Robotics, Porto Alegre, Brazil). The processing conditions applied were as follows: 20 kV voltage; 0.08 mL/min feed rate; 0.5 mm needle inner diameter; and 15 cm distance to collector. The process was performed at 25 °C. An aluminum plate (15 × 15 cm) was used to collect the nanofibers, which were dried overnight to eliminate residual solvent.

### 3.5. Thermogravimetric Analysis (TGA)

The thermal stability of nanofibers was evaluated using a thermogravimetric analyzer model Pyris 1 TGA (Perkin Elmer, San Jose, CA, USA). Samples were placed in platinum pans and heated from 25 to 800 °C at a rate of 10 °C/min under a nitrogen purge (20 mL/min).

### 3.6. Differential Scanning Calorimetry (DSC)

DSC studies were carried out using a DSC 8500 apparatus (Perkin Elmer, CA, USA). Samples of approximately 11 mg were loaded in aluminum pans and heated from 20 to 200 °C (10 °C/min heating rate) under a nitrogen flow of 20 mL/min.

### 3.7. Scanning Electron Microscopy (SEM)

The nanofibers were metallized with gold coating, and their morphological examination was performed using scanning electron microscopy (Zeiss EVO MA10 SEM, Oberkochen, Germany). Furthermore, energy dispersive X-ray spectroscopy (EDS, JEOL JXA-840, Tokyo, Japan) was applied to evaluate the presence of chemical elements within the nanofibers. The histograms presented here were acquired through the measurement of at least 100 nanofibers, coming from several images obtained through SEM, analyzed in at least four different regions of the material surface.

### 3.8. Fourier-Transform Infrared Spectroscopy (FTIR)

The chemical properties of the nanofibers were assessed through FTIR. The spectrum was obtained with the ATR method using a Perkin Elmer Spectrum IR Version 10.7.2, in the region between 4000 and 500 cm^−1^ at a resolution of 4 cm^−1^. Eight scans were performed for each spectrum [83].

### 3.9. Mechanical Analysis and Rheological Measurements

Information on the mechanical properties of the nanofibers, including tensile strength and elongation at break, were acquired using the TA.XT PLUS microcomputer-controlled texture analyzer (Stable Micro Systems, Godalming, UK). In accordance with ASTM D638, a 10 mm/min strain rate and a 40 mm clamping distance were used during the experiments. Nanofibers were cut into 50 × 10 mm samples, and the samples’ thickness was 0.1 mm. At least three repetitions were performed per sample, and the average values of these measurements were utilized.

The rheological properties of coacervates were examined in a Haake Mars III rheometer (Thermo Scientific, Dreieich, Germany). Viscosity was determined using a parallel plate rheometer (35 mm diameter, 1 mm gap), with the shear rate varying from 0.1 to 100 s^−1^ [84].

### 3.10. Antioxidant Activity

The antioxidant potential of nanofibers was assessed through the scavenging of 2,2-diphenyl-1-picrylhydrazyl (DPPH) [85] and 2,2′-azinobis-(3-ethylbenzothiazoline-6-sulfonic acid) (ABTS) radicals [86]. Both assays detect the ability of postulated antioxidants to neutralize the test radicals through electron transfer mechanisms. In both assays, the scavenging of DPPH or ABTS radicals was calculated as:

Scavenging (%) = [(Abs_control_ − Abs_sample_)/Abs_control_] × 100 (1)

Results were expressed as Trolox equivalents (TEAC), based on a Trolox calibration curve in the 0.1–2.0 mM range.

#### 3.10.1. DPPH Assays

The DPPH assays were performed with nanofibers and after nanofibers were submitted to an extraction protocol. In the first set of assays, the nanofibers (5 mg) were added to 1 mL of DPPH radical solution (60 µM, in methanol). Following homogenization, incubations were carried out for 30 min, 24 h, 40 h, and 48 h, in the dark and at room temperature. Afterwards, absorbance (Abs) was measured at 517 nm. Blank tests were prepared with distilled water replacing the samples [85].

In the second set of DPPH assays, nanofibers were extracted as previously recommended [66]. Briefly, nanofibers (5 mg) were dissolved in 4 mL of carbonate–bicarbonate buffer (0.2 M, pH 9.5) and vortexed for 3 min. Subsequently, the obtained solutions were treated in an ultrasonic bath (15 min; 40 kHz) and then centrifuged (12,800× *g*, 30 min, 20 °C). The resulting supernatant (50 µL) was mixed with 1 mL of 180 mM DPPH radical and, following 1 h of incubation (in the dark, at room temperature), the Abs was read at 517 nm.

#### 3.10.2. ABTS Assays

The ABTS assays were performed with nanofibers extracts, prepared as described above. The ABTS radical cation was obtained by mixing 5 mL of ABTS (7 mM) with 88 μL of K_2_S_2_O_8_ (140 mM). This mixture remained at room temperature, protected from light, for 12–16 h before use. The obtained radical solution was then diluted with phosphate-buffered saline (PBS; 5 mM, pH 7.4) until it reached an Abs of 0.700 (±0.02) at 734 nm. For the assays, suitably diluted nanofiber extracts (150 μL) were added to the ABTS radical solution (1 mL), and the Abs at 734 nm was read after 10 min [86]. Distilled water replaced the samples in control tests.

### 3.11. Hemolysis Test

Hemolytic activity was evaluated essentially as described elsewhere [87]. The cells were washed three times with PBS (900× *g*, 15 min, and 4 °C). The nanofibers were weighed (4 mg) by adding 1 mL of heparinized sheep blood (4%, *v*/*v*) and 1 mL of PBS (pH 7.4). The aliquots were incubated (60 min at 37 °C) and then centrifuged at 3000× *g* for 10 min. One mL of the supernatant was taken for reading on a spectrophotometer at 540 nm. Triton X-100 (0.1%, *v*/*v*) and PBS were used as positive and negative controls, respectively. Hemolytic activity was determined with the following equation:

Hemolytic activity (%) = (AS − AN)/(AP − AN) × 100(2)

where AS is the sample reading, AN is the negative control, and AP is the positive control.

## 4. Conclusions

This study describes the incorporation of an antioxidant keratin-based hydrolysate into polymeric nanofibers. It was possible to obtain nanofibrous materials with interesting antioxidant potential through the electrospinning technique, exploring the potential of a microbial bioprocess to obtain KH. The resulting nanofibers presented a homogeneous morphology and good thermal and mechanical properties, confirming that the formulations are suitable to produce bioactive nanofiber mats. Besides their antioxidant activity, these nanostructures showed a very low hemolysis rate indicating their potential as biocompatible materials. The successful production of nanofibrous scaffolds with remarkable antioxidant capacity suggests possible applications in regenerative medicine specifically through tissue engineering and mucoadhesive abilities. Especially, in the area of wound healing, nanofibrous scaffolds can be of great value since the inflammatory process starts instantly in a tissue damaged by injury, and these antioxidant nanomaterials can neutralize the effects of free radicals, as demonstrated by in vitro tests. Thus, future studies will be aimed at the in vivo assessment of biological activity and detailed toxicological investigation of these nanobiomaterials.

## Figures and Tables

**Table 1:** DSC thermal data for PCL nanofibers with the addition of 1%, 2.5%, or 5% KH ^1^.

Nanofiber	*T*m (°C)	Δ*H*m (J/g)	*T*onset (°C)	*X*c (%)
PCL 10% (control)	58.99	71.7060	54.89	87.87
PCL10 + 1% KH	57.92	47.7180	54.22	58.48
PCL10 + 2.5% KH	58.40	33.8602	54.40	41.49
PCL10 + 5% KH	58.74	66.3929	54.33	81.36
PCL 15% (control)	59.69	70.9667	55.08	86.97
PCL15 + 1% KH	57.88	45.5125	53.79	55.77
PCL15 + 2.5% KH	58.72	67.6502	54.48	82.90
PCL15 + 5% KH	58.09	51.8343	54.62	63.52

^1^ *T*m: melting temperature; Δ*H*m: enthalpy of fusion; *T*onset: onset of melting temperature; *X*c: crystallinity, calculated by *X*c = Δ*H*m/Δ*H*c, considering that the melting enthalpy of 100% crystalline PCL is 81.6 J/g.

**Table 2:** Mechanical properties of poly-ε-caprolactone (PCL 10% and PCL 15%) nanofibers. Nanofibers functionalized with feather keratin hydrolysate (KH) at different concentrations (1, 2.5, and 5% KH) were evaluated ^1^.

Nanofiber	Young’s Modulus (MPa)	Tensile Strength (MPa)	Elongation at Break (%)
PCL 10% (control)	42.64 ± 5.68	5.76 ± 0.17	138 ± 0.029
PCL10 + 1% KH	27.96 ± 2.81	1.43 ± 0.36	132 ± 0.054
PCL10 + 2.5% KH	25.93 ± 2.23	1.98 ± 0.40	142 ± 0.126
PCL10 + 5% KH	25.85 ± 3.69	0.84 ± 0.20	122 ± 0.026
PCL 15% (control)	24.81 ± 2.12	1.82 ± 0.44	183 ± 0.23
PCL15 + 1% KH	20.57 ± 1.23	1.59 ± 0.18	179 ± 0.052
PCL15 + 2.5% KH	18.99 ± 0.18	1.34 ± 0.059	199 ± 0.050
PCL15 + 5% KH	19.96 ± 0.59	1.30 ± 0.20	186 ± 0.038

^1^ Values are the means and standard deviations of three independent samples.

**Table 3:** Viscosity of polymeric solutions of 10% and 15% PCL loaded with 5% bioactive keratin hydrolysate (KH). The samples were compared with the respective controls (polymeric solutions without the KH).

Nanofiber	Absolute Viscosity η (mPas)	
	Control	5% KH
PCL 10%	25.82	184.89
PCL 15%	26.20	234.36

**Table 4:** Antioxidant properties of poly-ε-caprolactone (15% PCL) nanofibers ^1^.

Nanofiber	DPPH (%)	DPPH (μM) ^2^	ABTS (%)	ABTS (μM) ^2^
PCL (control)	nd ^3^	nd	nd	nd
PCL + 1% KH	49.03 ± 0.02	972.2 ± 3.0	44.19 ± 0.02	972.0 ± 7.4
PCL + 2.5% KH	49.24 ± 0.07	982.2 ± 13.5	49.61 ± 0.01	1153.3 ± 44.4
PCL + 5% KH	49.07 ± 0.01	974.7 ± 1.5	56.21 ± 0.006	1228.7 ± 18.5

^1^ Nanofibers functionalized with bioactive keratin hydrolysate (KH) at different concentrations (1, 2.5, and 5% KH) were subjected to an extraction protocol and the extracts evaluated for antioxidant activity. Data represent the mean ± standard deviation of three independent experiments. ^2^ Values expressed as Trolox equivalents (TEAC). ^3^ nd = not detected.

## Data Availability

Data is contained within the article.
